# The association between body roundness index and osteoporosis in American adults: analysis from NHANES dataset

**DOI:** 10.3389/fnut.2024.1461540

**Published:** 2024-10-04

**Authors:** Xunmeng Zhang, Jiarong Liang, Hong Luo, Huanhuan Zhang, Jing Xiang, Lianjin Guo, Xuemin Zhu

**Affiliations:** ^1^The Fourth Affiliated Hospital of Guangzhou Medical University, Guangzhou, China; ^2^Longhu Street Community Health Service Center, Guangzhou, China; ^3^Shitan Town Sanjiang Health Center, Guangzhou, China

**Keywords:** body roundness index, osteoporosis, obesity, bone mineral density, cross-sectional study, NHANES

## Abstract

**Background:**

An innovative way to quantify obesity that appropriately captures levels of visceral and body fat is the Body Roundness Index (BRI). The purpose of this study is to look at the relationship between BRI and osteoporosis (OP) in adult Americans.

**Methods:**

This study utilized data from the National Health and Nutrition Examination Survey (NHANES) collected between 2007 and 2018. NHANES is a research program designed to assess the health and nutritional status of adults and children in the United States. It conducts surveys focusing on various populations and health-related topics. Logistic regression analysis was employed to investigate the relationship between BRI and OP, adjusting for various covariates. BRI was categorized into four levels to further explore the association trends between different BRI levels and OP, enhancing the robustness of the results. Using restricted cubic spline (RCS) analysis, the dose–response relationship between BRI and OP was illustrated. Subgroup analyses were also carried out to evaluate the consistency and robustness of the findings.

**Results:**

This study included 8,899 participants aged 50 years and older, among whom 763 had OP. BRI and the prevalence of OP were inversely correlated in the fully adjusted model (OR = 0.79, 95% CI: 0.69–0.86). The prevalence of OP considerably reduced with higher BRI levels when BRI was converted from a continuous to a categorical variable in comparison to the lowest BRI quartile. RCS analysis revealed an L-shaped negative correlation between BRI and OP prevalence, with a threshold effect analysis identifying a breakpoint at BRI = 5.29. Each unit increase in BRI to the left of this breakpoint was linked to a 36% decrease in the probability of OP (OR = 0.64, 95% CI: 0.57–0.72). Based on stratified factor subgroup analyses, it was shown that the negative correlation between BRI and OP persisted.

**Conclusion:**

In a large, representative sample of American adults, this study identified a significant negative correlation between BRI and the prevalence of OP. Specifically, as BRI increases, the prevalence of osteoporosis decreases. Maintaining an appropriate and healthy BRI level may play a critical role in the prevention of osteoporosis. Therefore, regular monitoring of BRI and the adoption of appropriate health measures are essential for reducing the risk of osteoporosis.

## 1 Introduction

Reduced bone mass, degradation of the microstructure of the bone, increased fragility of the bone, and an increased risk of fractures are the hallmarks of osteoporosis (OP), a systemic skeletal disease (1, 2). OP is particularly prevalent among elderly postmenopausal women, primarily due to estrogen deficiency (3), which leads to decreased bone density and subsequently reduced mechanical strength of bones, making them prone to fractures (4). An estimated 10 million people in the US have OP (5), and an additional 43 million people have inadequate bone density, according to study estimates (6). With the aging population, the direct annual cost of OP is projected to reach $25.3 billion by 2025 (7). OP not only significantly increases the risk of fractures but also leads to a marked decline in quality of life (8), increased mortality, and substantially higher healthcare costs (9), thereby making it a major global public health challenge (10). Therefore, minimizing the difficulties associated with OP requires early identification and prevention.

Traditionally, the Body Mass Index (BMI) has been widely used to assess obesity and related health risks. However, BMI’s limitation lies in its inability to distinguish between fat and muscle tissue (11), and its inability to accurately reflect fat distribution (12). The Body Roundness Index (BRI) is an emerging body composition indicator first proposed in 2013, which combines height and waist circumference to more accurately reflect body fat distribution (13), offering a new perspective for assessing body fat percentage and visceral fat proportion (14). The advantage of this measurement is that it focuses not just on overall weight but more precisely reflects fat distribution patterns (15), particularly abdominal fat accumulation (16), which is a key factor in increasing the risk of many chronic diseases. Previous studies have demonstrated that BRI possesses high sensitivity and specificity in assessing health risks such as cardiovascular disease, diabetes, and metabolic syndrome (17–19). There is also a link between metabolic syndrome and BMD (20). As an indicator reflecting visceral fat accumulation (21), BRI provides significant value in predicting and assessing the risks of these diseases. We used data from the National Health and Nutrition Examination Survey (NHANES) to examine the relationship between BRI levels and the prevalence of OP in adults 50 years of age and older in order to shed light on the possible role of BRI in OP. This information may help in the development of future preventative or therapeutic approaches for OP.

## 2 Methods

### 2.1 Survey description

The goal of the National Health and Nutrition Examination study (NHANES), a two-year cycle national study, is to systematically evaluate the health and nutritional status of the American people. The Ethics Review Board has authorized this study endeavor, and each participant gave written informed permission. Researchers can examine a variety of health patterns and correlations using the quantity of public health information these data give.

### 2.2 Study population

This study utilized data collected by NHANES from 2007 to 2018. Due to the lack of femur bone density data for the 2011–2012 and 2015–2016 cycles, these two cycles were excluded from the analysis. The following three requirements had to be met in order for participants to be included: (1) they had to be adults 50 years of age or older; (2) they had to provide complete bone density measurement data; and (3) they had to provide complete waist circumference and height data. The last set of chosen research participants were added for additional investigation once these criteria were applied.

### 2.3 Calculation of BRI

BRI is calculated by combining two key body measurements: height (BH) and waist circumference (WC) (14). These measurements were obtained by professionally trained medical technicians at Mobile Examination Centers (MEC). The formula for calculating BRI is as follows:


$$BRI=364.2-365.5\times \sqrt{1-{\left(\frac{WC\left(m\right)/2\pi }{0.5\times BH\left(m\right)}\right)}^{2.}}$$

### 2.4 Definition of osteoporosis

NHANES used dual-energy X-ray absorptiometry (DXA) to measure the proximal femur of participants throughout the 2007–2010, 2013–2014, and 2017–2018 cycles. Shepherd Laboratories reviewed and analysed each participant and model scan using standard radiographic techniques and study-specific protocols developed for NHANES. Hologic software APEX v4.0 (Hologic) was used to analyse the acquired proximal femur scans, and bone mineral density (BMD) was measured at the femoral rotor, inter-rotor region, femoral neck, and throughout the femur. According to the World Health Organization (WHO) recommendations, the T-Score was calculated using the following formula: T-Score = (BMD measurement - BMD reference)/standard deviation. The reference standard is the BMD of Non-Hispanic White women between the ages of 20 and 29 years. Participants were diagnosed with OP when the T-Score was less than −2.5 standard deviations at any given site (22).

### 2.5 Covariates

To comprehensively analyze the relationship between BRI and OP, this study included various covariates such as demographic characteristics, lifestyle factors, health conditions, and laboratory test results. Demographic characteristics encompassed age, sex, race, poverty income ratio (PIR), and education level. PIR is divided into three levels: <1, 1 to <3, and ≥ 3 (23). It is computed by dividing total family income by the poverty line. Lifestyle variables encompassed smoking and physical activity. More than 100 cigarettes smoked throughout one’s lifetime was considered smoking. Physical activity was assessed using the Global Physical Activity Questionnaire to calculate metabolic equivalents (MET), with the formula: MET (minutes/week) = MET value × weekly frequency × duration of each activity (24). A MET value of less than 600 min per week was defined as inactivity. Health condition variables included chronic kidney disease, hypertension, high cholesterol, and diabetes, as determined by physician diagnosis records or self-reports. Laboratory tests included serum uric acid (SUA), high-density lipoprotein cholesterol (HDL-C), blood urea nitrogen (BUN), total cholesterol (TC), alanine aminotransferase (ALT), aspartate aminotransferase (AST), calcium, and phosphorus. All laboratory parameters were measured using a Roche Cobas 6,000 analyzer (c501 module). More detailed information on the analyte methods, principles, and operational procedures can be found in the NHANES Laboratory Procedures Manual.

### 2.6 Statistical analysis

After identifying participants who meet the inclusion criteria, a descriptive analysis based on osteoporosis status was conducted. Continuous variables were expressed as means (standard deviation), and categorical variables as percentages. The 95% confidence intervals (CI) and odds ratios (OR) for the relationship between BRI and OP were determined using logistic regression analysis. To enhance the robustness of the study results, BRI was categorized into four levels to analyze association trends within different BRI ranges. To investigate the nonlinear relationship between BRI and OP, restricted cubic spline (RCS) analysis was utilized, and threshold effect analysis was utilized to identify important spots. Additionally, subgroup analyses based on demographic and lifestyle factors were conducted to further explore the potential relationship between BRI and OP. Finally, additional analyses of BMD in different femoral regions were performed to verify the robustness of the study results. The statistical significance threshold was set at *p* < 0.05 for all data analyses, which were carried out using the R program (version 4.2.3; https://www.R-project.org).

## 3 Results

### 3.1 Characteristics of study population

**Figure 1:**
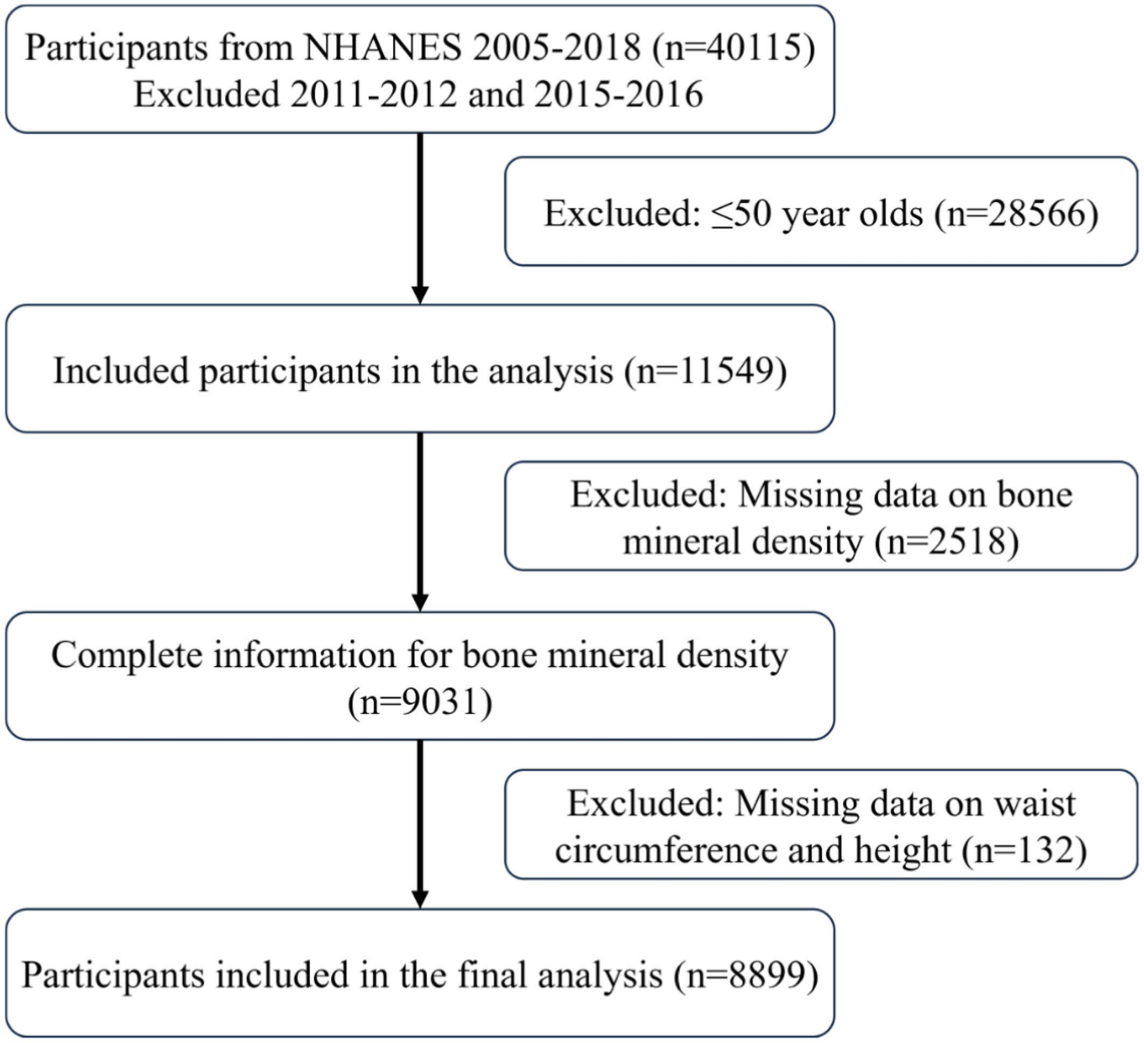
Include participants in the process.

Data from a total of 40,115 participants were extracted from the NHANES database. After the screening process (Figure 1), 8,899 participants were included in the subsequent study, comprising 8,136 non-osteoporotic participants and 763 osteoporotic participants. Baseline characteristics according to OP status are shown in Table 1. In contrast to the non-osteoporotic group, the osteoporotic group’s members exhibited lower levels of physical activity, were mostly Non-Hispanic White, had a greater proportion of females, and were typically older. They also exhibited higher levels of HDL-C, BUN, and serum phosphorus, along with lower waist circumference, height, BRI, and bone density in the femoral regions.

**Table 1:** Baseline characteristics of the study population.

Characteristic	Overall	Non-Osteoporosis	Osteoporosis	*p*-value
*n*	8,899	8,136	763	
Age (%)				<0.001
<65	4,873 (54.8)	4,670 (57.4)	203 (26.6)	
>65	4,026 (45.2)	3,466 (42.6)	560 (73.4)	
Sex (%)				<0.001
Female	4,317 (48.5)	3,788 (46.6)	529 (69.3)	
Male	4,582 (51.5)	4,348 (53.4)	234 (30.7)	
Race (%)				<0.001
Mexican American	1,178 (13.2)	1,103 (13.6)	75 (9.8)	
Non-Hispanic Black	1792 (20.1)	1729 (21.3)	63 (8.3)	
Non-Hispanic White	4,232 (47.6)	3,762 (46.2)	470 (61.6)	
Others	1,697 (19.1)	1,542 (19.0)	155 (20.3)	
Education level (%)				<0.001
Under high school	2,444 (27.5)	2,196 (27.0)	248 (32.5)	
High school or equivalent	2,129 (23.9)	1925 (23.7)	204 (26.7)	
Above high school	4,311 (48.4)	4,004 (49.2)	307 (40.2)	
PIR (%)				<0.001
<1	1,313 (16.5)	1,176 (16.2)	137 (20.1)	
1–3	3,490 (43.9)	3,127 (43.0)	363 (53.1)	
>3	3,147 (39.6)	2,964 (40.8)	183 (26.8)	
Activity status (%)				<0.001
Active	3,700 (41.6)	3,498 (43.0)	202 (26.5)	
Inactive	5,199 (58.4)	4,638 (57.0)	561 (73.5)	
Smoke (%)				0.014
No	4,428 (49.8)	4,016 (49.4)	412 (54.0)	
Yes	4,468 (50.2)	4,118 (50.6)	350 (45.9)	
Hypertension (%)				0.451
No	4,063 (45.7)	3,721 (45.7)	342 (44.8)	
Yes	4,822 (54.2)	4,401 (54.1)	421 (55.2)	
Hypercholesterolemia (%)				0.048
No	3,801 (45.8)	3,456 (45.5)	345 (48.7)	
Yes	4,432 (53.4)	4,079 (53.7)	353 (49.9)	
Diabetes (%)				0.171
No	6,813 (76.6)	6,210 (76.3)	603 (79.0)	
Yes	1762 (19.8)	1,623 (19.9)	139 (18.2)	
CKD (%)				<0.001
No	8,508 (95.6)	7,809 (96.0)	699 (91.6)	
Yes	374 (4.2)	313 (3.8)	61 (8.0)	
Total femur BMD (mean (SD)) (gm/cm^2^)	0.92 (0.16)	0.95 (0.15)	0.66 (0.09)	<0.001
Femoral neck BMD (mean (SD)) (gm/cm^2^)	0.76 (0.14)	0.78 (0.13)	0.53 (0.06)	<0.001
Trochanter BMD (mean (SD)) (gm/cm^2^)	0.70 (0.14)	0.72 (0.13)	0.50 (0.08)	<0.001
Intertrochanter BMD (mean (SD)) (gm/cm^2^)	1.10 (0.19)	1.13 (0.17)	0.79 (0.12)	<0.001
SUA (mean (SD)) (mg/dL)	5.65 (1.44)	5.68 (1.44)	5.31 (1.43)	<0.001
HDL (mean (SD)) (mmol/L)	1.40 (0.42)	1.38 (0.42)	1.54 (0.44)	<0.001
TC (mean (SD)) (mmol/L)	5.08 (1.11)	5.08 (1.11)	5.12 (1.13)	0.402
BUN (mean (SD)) (mmol/L)	5.60 (2.36)	5.54 (2.29)	6.19 (2.97)	<0.001
ALT (mean (SD)) (U/L)	23.78 (18.00)	24.14 (18.30)	19.84 (13.83)	<0.001
AST (mean (SD)) (U/L)	25.44 (13.67)	25.53 (13.89)	24.53 (11.02)	0.061
Calcium (mean (SD)) (mmol/L)	2.36 (0.10)	2.36 (0.09)	2.35 (0.11)	0.438
Phosphorus (mean (SD)) (mmol/L)	1.20 (0.18)	1.19 (0.18)	1.23 (0.18)	<0.001
WC (mean (SD)) (cm)	100.80 (14.02)	101.59 (13.78)	92.33 (13.78)	<0.001
Height (mean (SD)) (cm)	166.13 (10.11)	166.72 (9.97)	159.88 (9.48)	<0.001
BMI (mean (SD))	28.67 (5.70)	29.02 (5.65)	24.96 (4.86)	<0.001
BRI (mean (SD))	5.74 (1.95)	5.80 (1.95)	5.08 (1.88)	<0.001

Mean (SD) for continuous variables, % for categorical variables. ALT: Alanine Aminotransferase; AST: Aspartate Aminotransferase; BRI: Body roundness index; BUN: Blood urea nitrogen; CKD: Chronic kidney disease; HDL-C: High-density lipoprotein cholesterol; PIR, Poverty income ratio; SUA: Serum uric acid; TC: Total cholesterol; WC: Waist Circumference.

### 3.2 Association between BRI and prevalence of osteoporosis

Table 2 presents the results of the logistic regression analyses conducted to examine the relationship between WC, BMI, BRI and OP prevalence. It was found that WC, BMI, BRI were negatively associated with OP prevalence in model 1. The results remained stable with gradual adjustment of different covariates. After appropriate adjustment for all variables, the OP prevalence decreased by 5% (OR = 0.95, 95% CI: 0.94–0.96), 14% (OR = 0.86, 95% CI: 0.84–0.89), and 21% (OR = 0.79, 95% CI: 0.69–0.86) for each unit increase in WC, BMI, BRI, respectively. When BRI was converted from a continuous to a categorical variable, the prevalence of OP compared with the lowest BRI quartile declined substantially with increasing levels of BRI (p trend <0.001). This trend persisted even after adjustment for all covariates, suggesting a robust negative association between BRI and OP.

**Table 2:** The relationship between BRI and osteoporosis.

		Model 1 OR (95%CI) *p*-value	Model 2 OR (95%CI) *p*-value	Model 3 OR (95%CI) *p*-value
Osteoporosis	WC	0.95 (0.94, 0.96) <0.001	0.95 (0.94, 0.96) <0.001	0.95 (0.94, 0.96) <0.001
	BMI	0.86 (0.83, 0.88) <0.001	0.86 (0.84, 0.89) <0.001	0.86 (0.84, 0.89) <0.001
	BRI	0.82 (0.76, 0.88) <0.001	0.77 (0.72, 0.83) <0.001	0.79 (0.69, 0.86) <0.001
	Q1	[Reference]	[Reference]	[Reference]
	Q2	0.56 (0.44, 0.71) <0.001	0.53 (0.41, 0.69) <0.001	0.61 (0.46, 0.82) 0.002
	Q3	0.50 (0.38, 0.65) <0.001	0.41 (0.32, 0.54) <0.001	0.47 (0.32, 0.69) <0.001
	Q4	0.43 (0.33, 0.57) <0.001	0.33 (0.25, 0.43) <0.001	0.35 (0.26, 0.46) <0.001
	p for trend	<0.001	<0.001	<0.001

BRI: Body roundness index; OR: Odds Ratio; CI: Confidence Interval. Model 1: No covariates adjusted; Model 2: Adjusted for Age, Sex, and Race; Model 3: Adjusted for Age, Sex, Race, Educational level, PIR, SUA, HDL-C, TC, BUN, ALT, AST, Calcium, Phosphorus, Smoke, Hypertension, Hypercholesterolemia, CKD, Diabetes.

### 3.3 Analysis of nonlinear relationships and saturation effects

**Figure 2:**
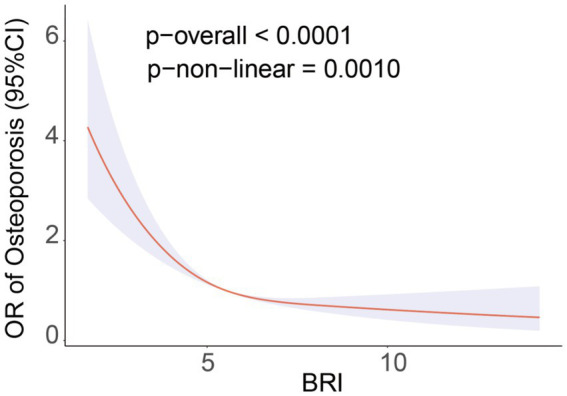
RCS curve fits the association of BRI with osteoporosis. Adjusted for Age, Sex, Race, Educational level, PIR, SUA, HDL-C, TC, BUN, ALT, AST, Calcium, Phosphorus, Smoke, Hypertension, Hypercholesterolemia, CKD, Diabetes.

The study using restricted cubic splines (RCS) showed a non-linear relationship between BRI and the prevalence of OS (P-non-linear = 0.0010). This relationship is defined by a negative correlation that has an L-shaped pattern (Figure 2). Threshold effect analysis identified a breakpoint at BRI = 5.29 for the OS group. Segmental logistic regression analysis revealed that for BRI values below 5.29, each 1-unit increase in BRI was linked to a 41% decrease in the probability of OS (odds ratio = 0.64, 95% confidence interval: 0.57–0.72). Nevertheless, as the BRI exceeds 5.29, the impact of increasing BRI on the occurrence of OS gradually decreases (OR=0.88, 95% CI: 0.81-0.96) (Table 3).

**Table 3:** Analysis of the BRI saturation effect and Osteoporosis.

	BRI (%)	OR (95%CI) p-value
Osteoporosis	Standard linear model	0.78 (0.73, 0.82) <0.001
	BRI < 5.29	0.64 (0.57, 0.72) <0.001
	BRI > 5.29	0.88 (0.81, 0.96) <0.001
	Log-likelihood ratio test	<0.001

BRI: Body roundness index; OR: Odds Ratio; CI: Confidence Interval. Model 3: Adjusted for Age, Sex, Race, Educational level, PIR, SUA, HDL-C, TC, BUN, ALT, AST, Calcium, Phosphorus, Smoke, Hypertension, Hypercholesterolemia, CKD, Diabetes.

### 3.4 Subgroup analysis

**Figure 3:**
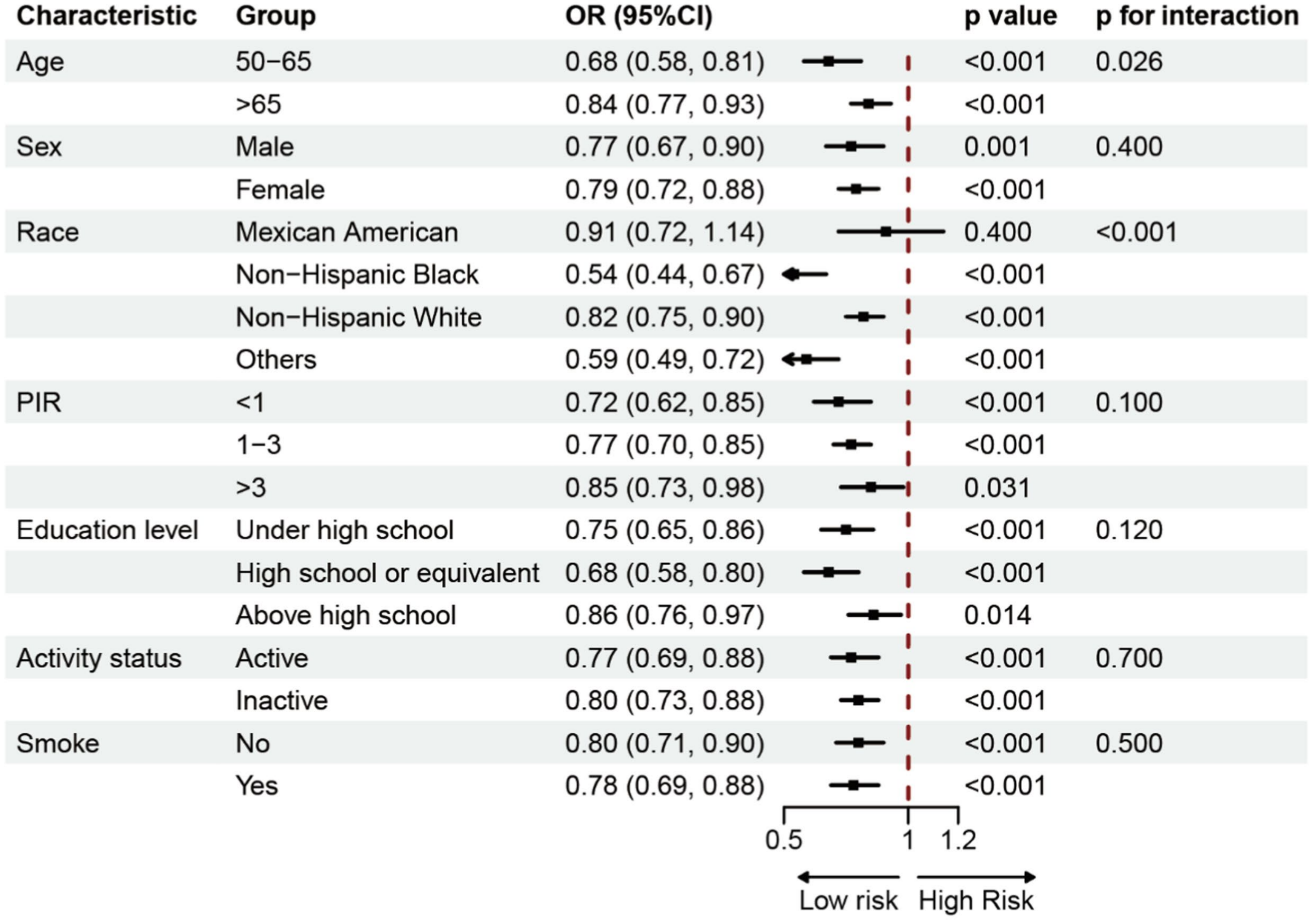
Subgroup analysis of the association between BRI and Osteoporosis.

In order to investigate the possible correlation between BRI and OP, a subgroup analysis was performed. This analysis involved categorizing individuals based on parameters such as age, sex, race, poverty income ratio (PIR), education level, physical activity, and smoking status. The study was conducted alongside Model 3 (Figure 3). The findings demonstrated a consistent negative correlation between BRI and the prevalence of OP across various groups.

## 4 Discussion

In 8,899 subjects, this cross-sectional study sought to determine if BRI and OP prevalence were related. The findings showed an L-shaped nonlinear association with a negative link between BRI and OP prevalence. The OP breakpoint was determined via threshold effect analysis to be BRI = 5.29. The chance of OP was shown to decrease by 41% for every unit increase in BRI until to this breakpoint (OR = 0.64, 95% CI: 0.57–0.72). However, after the breakpoint, the effect of increasing BRI on OP prevalence progressively decreased. Finally, subgroup analysis further confirmed the robustness of these findings. Therefore, these findings suggest that higher BRI levels are associated with a reduced risk of OP among American adults and that BRI is an independent protective factor against OP.

As far as we are aware, this is the first research to assess the relationship between BRI and OP. The BRI is a brand-new obesity index that precisely measures levels of visceral and body fat (25). In this study, BRI was negatively correlated with the prevalence of OP. This finding aligns with previous research suggesting that moderate weight gain has a protective effect on bone density (26, 27). A 13-year descriptive study of individuals aged 60 and above, using the Visceral Adiposity Index (VAI) to reflect abdominal fat distribution, found that when VAI increased to a threshold of 0.68 g/cm^2^, femoral BMD no longer increased and might even decrease (28). This implies that moderate visceral fat accumulation may positively impact bone health. Similarly, a cross-sectional study involving 6,143 adolescents aged 8–19 years found a significant positive and saturation correlation between BMI and BMD [0.014 (0.013, 0.014)] (29). For each unit increase in BMI, total BMD increased by 0.014 g/cm^2^, indicating a saturation effect as well. Another observational study involving 4,056 participants demonstrated a positive saturation effect of waist circumference (WC) at 70.5 cm on BMD, suggesting that moderate obesity may promote better bone mass development in adolescents (30). Furthermore, several studies have found a positive correlation between obesity and both bone density and bone metabolism, implying that a moderate amount of fat is crucial for effectively managing bone metabolic health (31, 32). This study further reveals a nonlinear relationship between BRI and OP, showing that within a lower BRI range, increases in BRI significantly reduce the risk of OP, but beyond a certain threshold, this protective effect diminishes. This finding suggests that moderate fat distribution may positively impact bone health by increasing mechanical load on bones and promoting the secretion of bone-forming factors (33–35). However, excessive obesity may negatively affect bone health through various mechanisms, such as the secretion of inflammatory factors and hormones by adipose tissue, potentially leading to OP.

Multiple potential mechanisms are associated with the development of OP, but the underlying mechanisms linking BRI and OP remain unclear. The negative correlation between BRI and OP may involve several mechanisms. First, moderate fat distribution can increase mechanical load on bones, thereby stimulating bone formation (36). Bone is a dynamic tissue whose structure and density are significantly influenced by mechanical loading (37–39). Osteocytes, located within the bone matrix, may sense mechanical load changes through a mechanosensitive ion channel, Piezo1 (40), and generate signals that alter osteoblast bone formation (41). Moderate weight gain can promote bone formation and mineralization by increasing mechanical load on bones. Second, adipose tissue can secrete various hormones and cytokines (42), such as leptin, adiponectin, and inflammatory factors (43–45), which can influence bone metabolism and bone density (46). Receptor activation of NF-κB ligand (RANKL) expression is increased by leptin, which simultaneously promotes osteoclast differentiation and limits osteoblast proliferation via activating the molecular clock and Ap-1 gene in osteoblasts (47). Another pathway involves hypothalamic neuropeptide (cocaine-and amphetamine-regulated transcript) CART inhibiting RANKL expression in osteoblasts, thus inhibiting osteoclast differentiation and increasing bone density (34). Nevertheless, adiponectin possesses anti-inflammatory qualities (48), which decrease pro-inflammatory cytokine production by blocking NF-κB signaling and activating the AMP-activated protein kinase (AMPK) pathway, therefore lowering inflammation-mediated bone resorption (49).

Our study has several strengths. It is the first to evaluate the relationship between BRI and the risk of OP in American adults. The relatively large sample size allows for more accurate and consistent estimates. BRI is superior to traditional obesity indices in assessing individual fat distribution. Our research shows a consistent positive correlation between BRI and OP prevalence, proving that this is not an accident. Additionally, we adjusted for confounding variables based on demographic characteristics and chronic disease conditions. Furthermore, we conducted stratified subgroup analyses to investigate the relationship between BRI and OP in different populations, suggesting the need for more precise OP prevention strategies. Our study does, however, have several shortcomings. First, the causal relationship between BRI and OP cannot be established by the cross-sectional design. To confirm these results and investigate the possible use of BRI in the management and prevention of OP, more long-term research is required. Second, although our study controlled for various covariates, there may still be unmeasured confounding factors, such as dietary habits, physical activity intensity, and medication use, that could affect the results. Finally, as a relatively new body composition index, BRI’s clinical application and widespread use require further research support.

## 5 Conclusion

In a large, representative sample of American adults, this study identified a significant negative correlation between BRI and the prevalence of OP. Specifically, as BRI increases, the prevalence of osteoporosis decreases. Maintaining an appropriate and healthy BRI level may play a critical role in the prevention of osteoporosis. Therefore, regular monitoring of BRI and the adoption of appropriate health measures are essential for reducing the risk of osteoporosis.

## Data Availability

Publicly available datasets were analyzed in this study. This data can be found at: https://www.cdc.gov/nchs/nhanes/nhanes.
